# Supplementary material for: A Novel Screen for Expression Regulators of the Telomeric Protein TRF2 Identified Small Molecules That Impair TRF2 Dependent Immunosuppression and Tumor Growth
Source: Cancers (Basel). 2021 Jun 15;13(12):2998. doi: 10.3390/cancers13122998 (PMC8232760; doi:10.3390/cancers13122998)
Supplement: Supplementary file 1 [file cancers-13-02998-s001.zip › 8_El Mai et al_TableS1.pdf]

Table S1 : **Summary of TRF2 drug screening experiments. A.** Hits from the primary screening using flow cytometry after treatment with 10 µM drugs as described in Figure S1D ("+" : upregulation of TRF2; "-": downregulation of TRF2). **B.** Hits from the secondary screening using flow cytometry after treatment with 10 µM drugs as described in Figure S1E ("+" : upregulation of TRF2; "-": downregulation of TRF2). **C.** Analysis from western blot screening after treatment with 10 µM drugs as described in Figure S2 ("+" : upregulation of TRF2; "nc": no changes on TRF2 levels; "-": downregulation of TRF2).

Targetted Signaling Pathways	Drug number	Drug name	Hits from the primary screening (a)	Hits from the secondary screening (b)	Western blot analysis (c)
Control	1	Gemcitabin	-	-	-
Ion ligands	2	Amlodipine			
	3	(±)-U-50488			
	4	(±)-Verapamil·HCl			
	5	(R)-(+)-Bay K 8644			
	6	4-Aminopyridine			
	7	5-Hydroxydecanoic acid			
	8	Aconitine	+		
	9	AM 92016			
	10	Amantidine·HCl			
	11	Amiloride·HCl			
	12	Amiodarone			
	13	Amlodipine			
	14	Antibiotic A-23187	-		
	15	Benzamil			
	16	Bepridil			
	17	BTP2			
	18	Cyclopiazonic acid	-		
	19	Dantrolene			
	20	Diazoxide			
	21	Dichlorobenzamil			
	22	Diltiazem acid			
	23	Diltiazem·HCl			
	24	E-4031			
	25	Fipronil			
	26	Flecainide acetate			
	27	FLP			
	28	Flufenamic acid	+		
	29	Flunarizine·2HCl			
	30	Fluspirilene			
	31	FPL-64176			
	32	Gingerol			
	33	Glipizide			
	34	Glyburide			
	35	Grayanotoxin III			
	36	IAA-94	+		
	37	ISRA			
	38	L-(-)-cis-Diltiazem·HCl			
	39	Lidocaine·HCl·H_2O	+		
	40	Loperamide·HCl			
	41	Methoxyverapamil			
	42	Minoxidil			
	43	Minoxidil sulfate			
	44	Nicardipine·HCl			
	45	Nifedipine			
	46	Nifedipine			
	47	Niflumic acid			
	48	Niguldipine			
	49	Nimodipine			
	50	Nitrendipine			

	51	N-phenylanthranilic acid			
	52	NPPB	+		
	53	NS-1619			
	54	Paxilline	+		
	55	PCO-400			
	56	Penitrem A	+	+	nc
	57	Phenamil	+		
	58	Phentolamine HCl	+		
	59	Phenytoin			
	60	Pimozide			
	61	Pinacidil H_2O	+		
	62	Procainamide·HCl			
	63	Propafenone	+		
	64	Quinidine·HCl·H_2O	+		
	65	Quinine HCl 2H_2O	+		
	66	QX-314 bromide	+		
	67	Ryanodine			
	68	SDZ-201106			
	69	SKF-96365	+		
	70	SR			
	71	Tetrandrine			
	72	Thapsigargin	-		
	73	TMB-8·HCl			
	74	Tolazamide			
	75	Tolbutamide			
	76	U-37883A·HCl	-		
	77	Veratridine	+		
	78	Veridine			
	79	YS035			
	80	ZAPB	+		
	81	ZM226600			
Phosphatase Inhibitors	82	9,10-Phenanthrenequinone			
	83	Alendronate			
	84	Alexidine·2HCl	-	-	-
	85	B4-Rhodanine			
	86	Benzylphosphonic acid			
	87	BML-260			
	88	BML-267			
	89	BML-267 Ester			
	90	BN-82002			
	91	BVT-948	-	-	-
	92	Cantharidic acid	-		
	93	Cantharidin	-		
	94	CinnGel			
	95	Cyclosporin A			
	96	Cypermethrin			
	97	Deltamethrin			
	98	Endothall			
	99	Fenvalerate			
	100	Gossypol	-		
	101	Levamisole HCl			
	102	L-p-Bromotetramisole oxalate			
	103	NSC-663284	-		
	104	NSC-95397			
	105	OBA			
	106	OBA Ester			
	107	Pentamidine	-		
	108	RK-682			
	109	RWJ-60475			
	110	RWJ-60475 (AM)3			
	111	Sanguinarine chloride			
	112	Shikonin			
	113	Tetramisole HCl			

	114	Tyrphostin 8			
Kinase	115	dimethyl ether			
	116	2-Aminopurine			
	117	2-Hydroxy-5-(2,5-dihydroxybenzylamino)benzoic acid			
	118	5,6-dichloro-1-ß-D-ribofuranosylbenzimidazole			
	119	5-Iodotubericidin			
	120	AG			
	121	AG-126			
	122	AG-1296			
	123	AG-370			
	124	AG-490			
	125	AG-494			
	126	AG-825			
	127	AG-879	-		
	128	Apigenin			
	129	BAY 11-7082			
	130	BML-257			
	131	BML-259			
	132	BML-265	-		
	133	Daidzein			
	134	D-erythro-Sphingosine			
	135	Erbstatin analog			
	136	Genistein			
	137	GF 109203X			
	138	GW 5074			
	139	H-7·2HCl	-		
	140	H-8			
	141	H-89·2HCl			
	142	H-9·HCl	-		
	143	HA-1004·2HCl			
	144	HA-1077·2HCl			
	145	Hydroxy-2-naphthalenylmethylphosphonic acid			
	146	Hypericin			
	147	Indirubin			
	148	Indirubin-3'-monooxime			
	149	IRESSA	-	-	-
	150	Iso-olomoucine			
	151	Kenpaullone			
	152	KN-62	-		
	153	KN-93	-		
	154	Lavendustin A			
	155	LFM-A13			
	156	LY 294002	-		
	157	ML-7·HCl			
	158	ML-9·HCl			
	159	N9-isopropyl-olomoucine			
	160	Olomoucine			
	161	Palmitoyl-DL-carnitine			
	162	PD-98059			
	163	Piceatannol			
	164	PKC-412			
	165	PP1			
	166	PP2			
	167	Quercetin·$2H_2O$			
	168	Rapamycin			
	169	RG-1462			
	170	Ro 31-8220 mesylate			
	171	Roscovitine			
	172	Rottlerin	-		
	173	Rx			
	174	SB-202190			
	175	SB-203580			
	176	SC-514			

	177	SP 600125			
	178	Staurosporine			
	179	SU 4312			
	180	SU1498			
	181	SU-5402			
	182	SU6656	+		
	183	Terreic acid			
	184	Triciribine	-	-	-
	185	TYRPHOSTIN 1			
	186	TYRPHOSTIN 23			
	187	TYRPHOSTIN 25			
	188	TYRPHOSTIN 46			
	189	TYRPHOSTIN 47			
	190	TYRPHOSTIN 51			
	191	TYRPHOSTIN 9	-	-	-
	192	TYRPHOSTIN AG 1288			
	193	TYRPHOSTIN AG 1295			
	194	TYRPHOSTIN AG 1478			
	195	U0126			
	196	U-0126	-		
	197	Wortmannin	-		
	198	Y27			
	199	Y-27632·2HCl			
	200	ZM 336372			
	201	ZM 449829			
Epigenetics	202	2,4-Pyridinedicarboxylic Acid			
	203	5-Aza-2'-deoxycytidine			
	204	AGK2			
	205	Aminoresveratrol sulfate			
	206	Anacardic acid			
	207	Apicidin			
	208	B2			
	209	BIX-01294	-		
	210	BML-210			
	211	BML-266			
	212	BML-278			
	213	BML-281			
	214	Butyrolactone 3			
	215	CI-994			
	216	CTPB			
	217	EX-527			
	218	Fluoro-SAHA			
	219	Garcinol	-	-	-
	220	Isonicotinamide			
	221	ITSA-1			
	222	M-344			
	223	MC-1293			
	224	NCH-51			
	225	Nicotinamide			
	226	NSC-3852			
	227	Nullscript			
	228	Oxamflatin			
	229	Phenylbutyrate·Na			
	230	Piceatannol			
	231	Resveratrol	-		
	232	SAHA			
	233	Salermide			
	234	Scriptaid			
	235	Sirtinol			
	236	Splitomicin	-		
	237	Suberoyl bis-hydroxamic acid			
	238	Suramin·6Na			
	239	Tranylcypromine hemisulfate			

	240	Triacetylresveratrol			
	241	Trichostatin A			
	242	Valproic acid			
	243	Valproic acid hydroxamate			
	244	Zebularine			
Nuclear Receptor ligands	245	13(S)-Hydroxy-9Z,11E-octadecadienoic acid			
	246	13-cis Retinoic acid			
	247	13-cis-Retinol			
	248	15-Deoxy-D12,14-prostaglandin J2			
	249	17b-Estradiol			
	250	1a,25-Dihydroxyvitamin D3			
	251	24(S),25-Epoxycholesterol	-		
	252	24(S)-Hydroxycholesterol	-		
	253	25-Hydroxyvitamin D3			
	254	3,5-Diiodo-4-hydroxyphenylpropionic acid			
	255	3,5-Diiodo-L-thyronine			
	256	3,5-Diiodo-L-tyrosine			
	257	3a, 5a-Androstanol			
	258	3a, 5a-Androstenol			
	259	3-Methylcholanthrene	+		
	260	4-Hydroxyphenylretinamide			
	261	4-Hydroxyretinoic acid	+		
	262	5,8,11,14-Eicosatetraynoic acid			
	263	5b-Pregnan-3,20-dione			
	264	6a-Fluorotestosterone			
	265	6-Formylindolo [3,2-B] carbazole			
	266	9-cis Retinoic acid			
	267	Acetyl-S-farnesyl-L-cysteine			
	268	Acitretin			
	269	Adapalene			
	270	AGC (Acetyl-geranyl-cysteine)			
	271	all-trans-Retinol	+		
	272	AM-580			
	273	Androstenedione			
	274	BADGE			
	275	Bezafibrate			
	276	Carbacyclin			
	277	Chenodeoxycholic acid			
	278	Cholic acid			
	279	Ciglitazone			
	280	CITCO			
	281	Clofibric acid			
	282	Cortisone			
	283	Deoxycholic acid			
	284	Dexamethasone	+	+	+
	285	Diindolylmethane			
	286	Docosa-4Z,7Z,10Z,13Z,16Z,19Z-hexaenoic acid	+		
	287	Estrone			
	288	Farnesol			
	289	Farnesylthioacetic acid			
	290	Gemfibrozil			
	291	Geranylgeraniol			
	292	Glycocholic acid			
	293	Glycodeoxycholic acid			
	294	GW 7647			
	295	GW 9662			
	296	GW4064			
	297	Lithocholic acid			
	298	LY 171883			
	299	Methoprene acid			
	300	Mifepristone	+	+	nc
	301	N-Acetyl-S-geranygeranyl-L-cysteine			
	302	N-Oleoylethanolamide			

	303	Paxilline	+	+	nc
	304	Pioglitazone	+		
	305	Pregnenolone			
	306	Pregnenolone-16(alpha)-carbonitrile			
	307	Progesterone			
	308	Retinoic acid, all trans			
	309	Retinyl acetate			
	310	Rifampicin			
	311	S-Farnesyl-L-cysteine methyl ester			
	312	Tamoxifen			
	313	Taurocholic acid			
	314	Taurodeoxycholic acid			
	315	TCPOBOP			
	316	Tetradecylthioacetic acid			
	317	Troglitazone			
	318	TTNPB			
	319	WY-14643			
	320	Z-Guggulsterone	+	+	nc
Wnt pathway	321	(-)-Terreic acid			
	322	1-Azakenpaullone			
	323	3253-5986			
	324	5-Aza-2-deoxycytidine(Decitabine)			
	325	Anandamide			
	326	Apigenin			
	327	AR-A014418	-	-	-
	328	Bafilomycin A1	-	-	nc
	329	BIO			
	330	BML-284			
	331	BML285 (Diaminoquinazoline)	-		
	332	BML286 (3289-8625)			
	333	Bosutinib (SKI-606)	-	-	-
	334	Box5			
	335	Cardamonin			
	336	Carnosol			
	337	CCT036477			
	338	Celecoxib			
	339	CHIR99021	-		
	340	Curcumin	-		
	341	D4476	-		
	342	Deoxycholic acid			
	343	DHA			
	344	Diarylsulfonesulfonamide			
	345	Diclofenac·Na			
	346	Doxorubicin·HCl (3013-0085)			
	347	EGCG			
	348	Ellagic Acid			
	349	Endo IWR1			
	350	EPA			
	351	Exifone			
	352	Flavanone			
	353	Forskolin			
	354	Foxy-5			
	355	Gallic acid			
	356	Genistein			
	357	GW9662			
	358	Harmine·HCl			
	359	Hexachlorophene	-	-	nc
	360	ICG-001	-	-	nc
	361	IM-12			
	362	Imatinib mesylate	-		
	363	IQ1			
	364	IWP-2			
	365	IWR-1			

	366	JS-K	-		
	367	Kenpaullone			
	368	LY456236·HCl			
	369	NCI16221			
	370	Niclosamide	-	-	-
	371	NO-ASA			
	372	PGE2			
	373	PNU74654			
	374	PNU-74654			
	375	PP2	-		
	376	Pterostilbene			
	377	Purpurogallin			
	378	Pyrvinium pamoate			
	379	QS-11	+	+	nc
	380	Quercetin			
	381	Resveratrol	-		
	382	Retinoic Acid			
	383	Riluzole			
	384	Rosiglitazone maleate			
	385	SB-216763			
	386	Sodium valproate			
	387	Sulindac			
	388	Sulindac Sulfide			
	389	Thalidomide			
	390	TNP-470	+		
	391	Trichostatin A	+		
	392	Troglitazone			
	393	Usnic acid			
	394	Val-Val-Val			
	395	WAY-262611	-		
	396	XAV939			
