# Supplementary figures and images for: A Novel Screen for Expression Regulators of the Telomeric Protein TRF2 Identified Small Molecules That Impair TRF2 Dependent Immunosuppression and Tumor Growth
Source: Cancers (Basel). 2021 Jun 15;13(12):2998. doi: 10.3390/cancers13122998 (PMC8232760; doi:10.3390/cancers13122998)

Figure S1

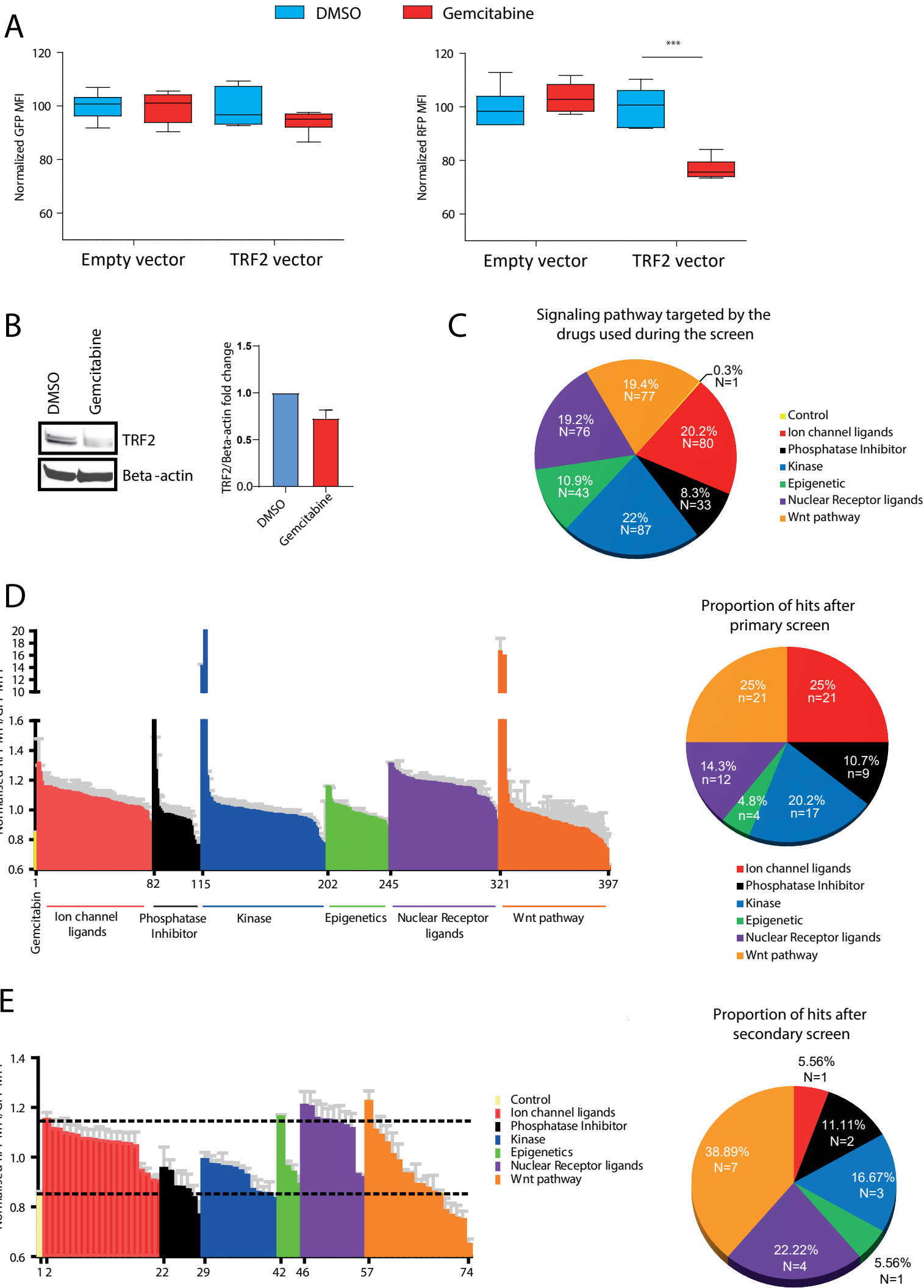

Supplement: Supplementary file 1 [file cancers-13-02998-s001.zip › 4_El Mai et al_FigureS1.pdf]

Figure S2

A

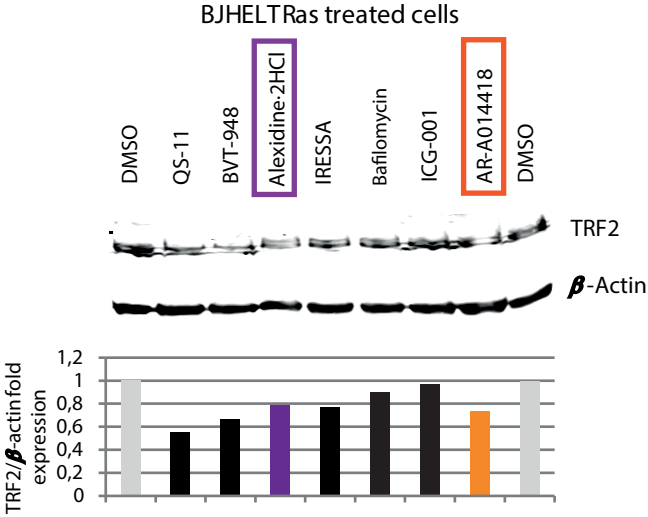

B

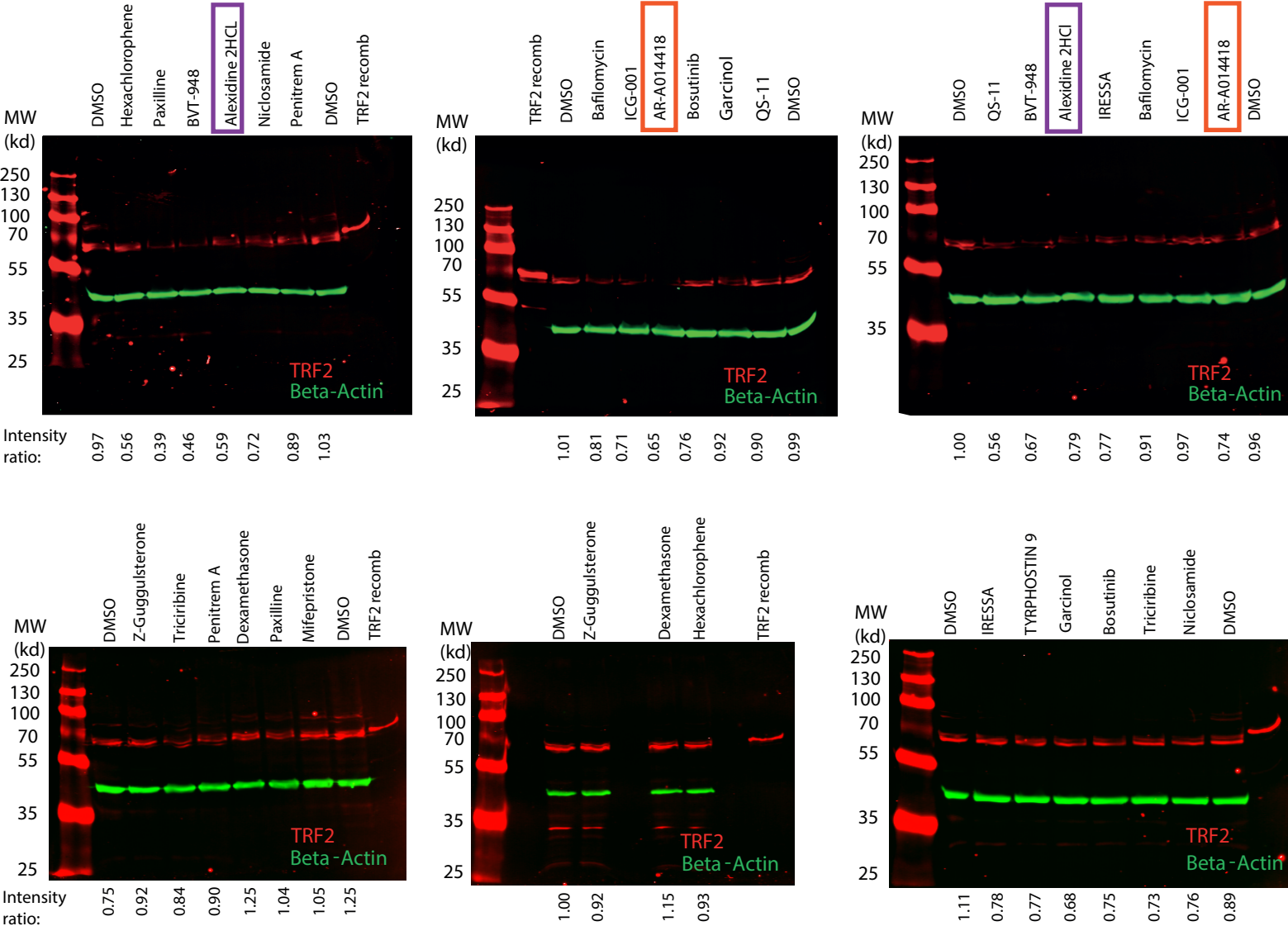

Supplement: Supplementary file 1 [file cancers-13-02998-s001.zip › 5_El Mai et al_FigureS2.pdf]

Figure S3

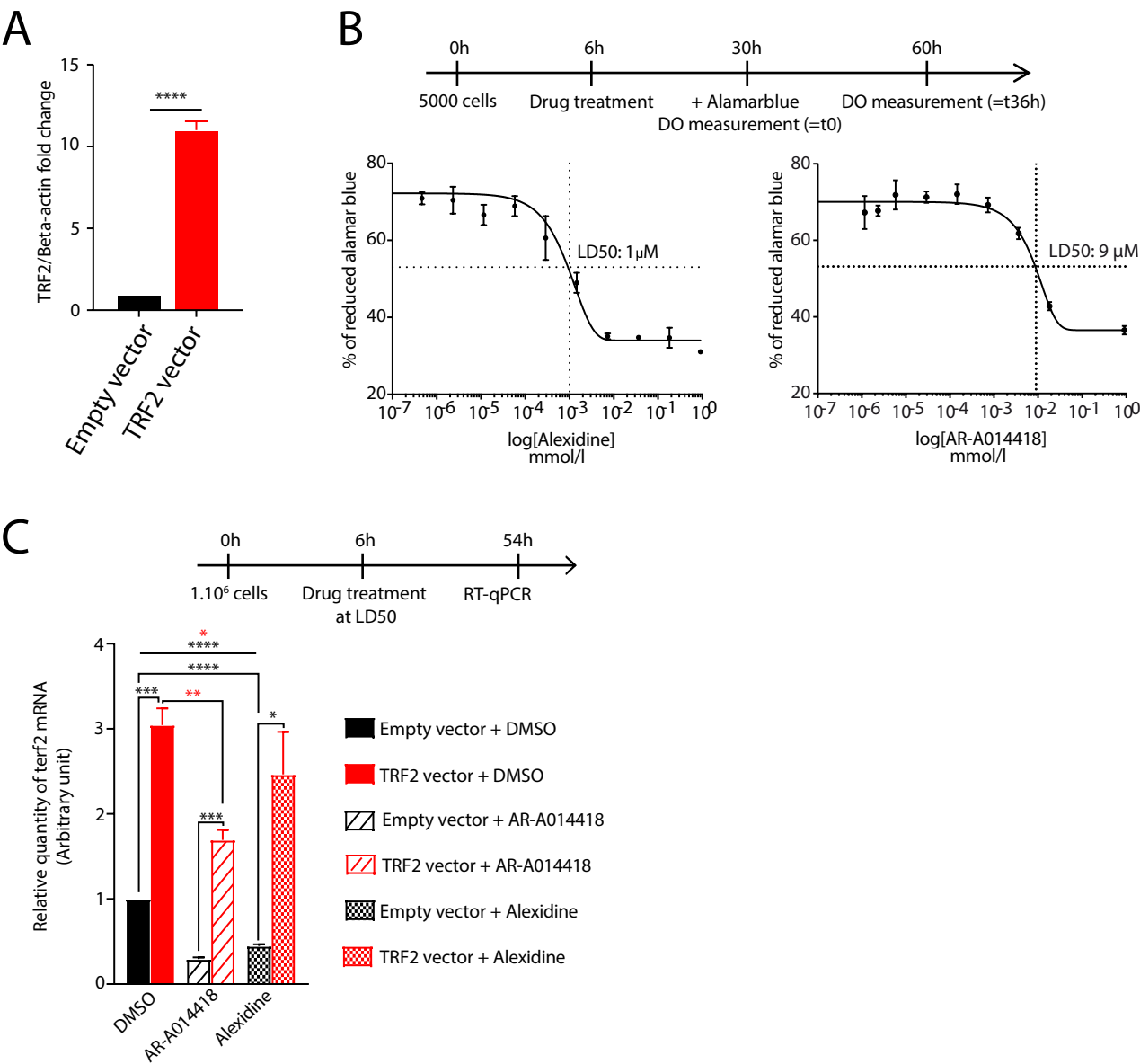

Supplement: Supplementary file 1 [file cancers-13-02998-s001.zip › 6_El Mai et al_FigureS3.pdf]

Figure E4

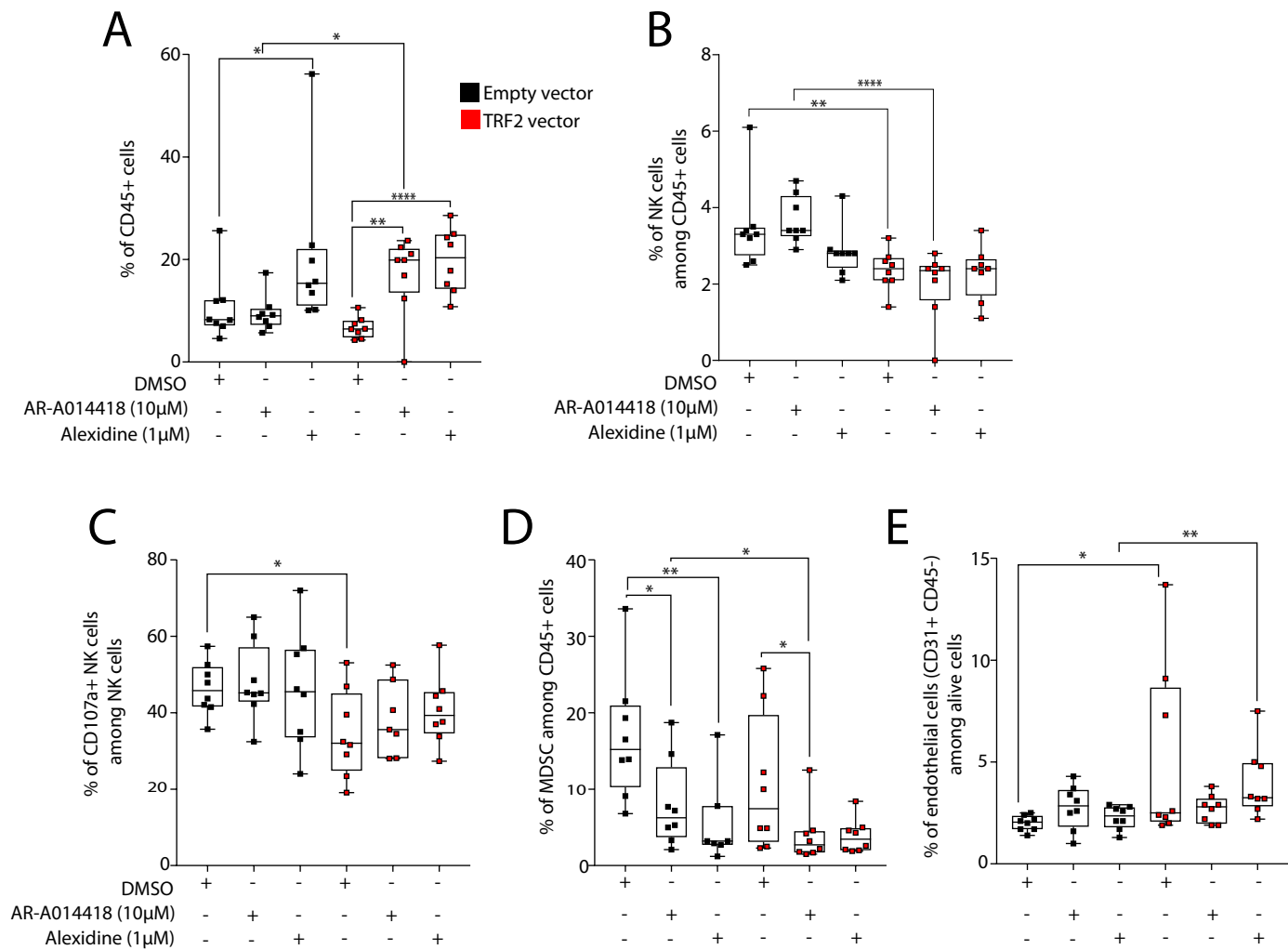

Supplement: Supplementary file 1 [file cancers-13-02998-s001.zip › 7_El Mai et al_FigureS4.pdf]

A

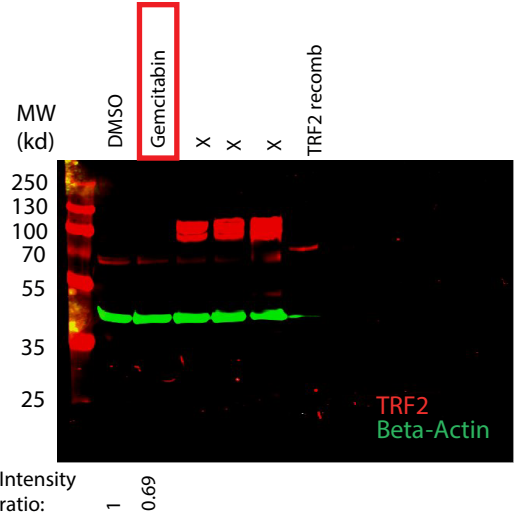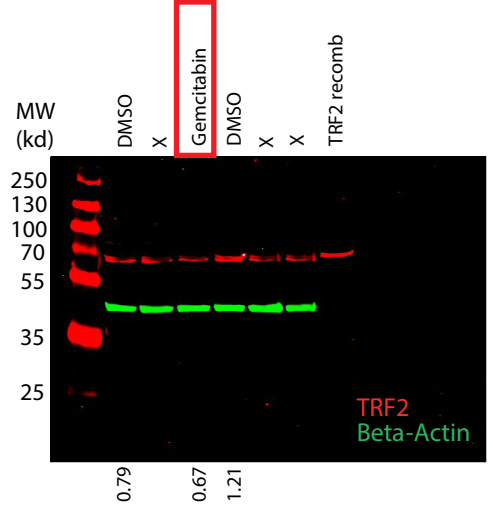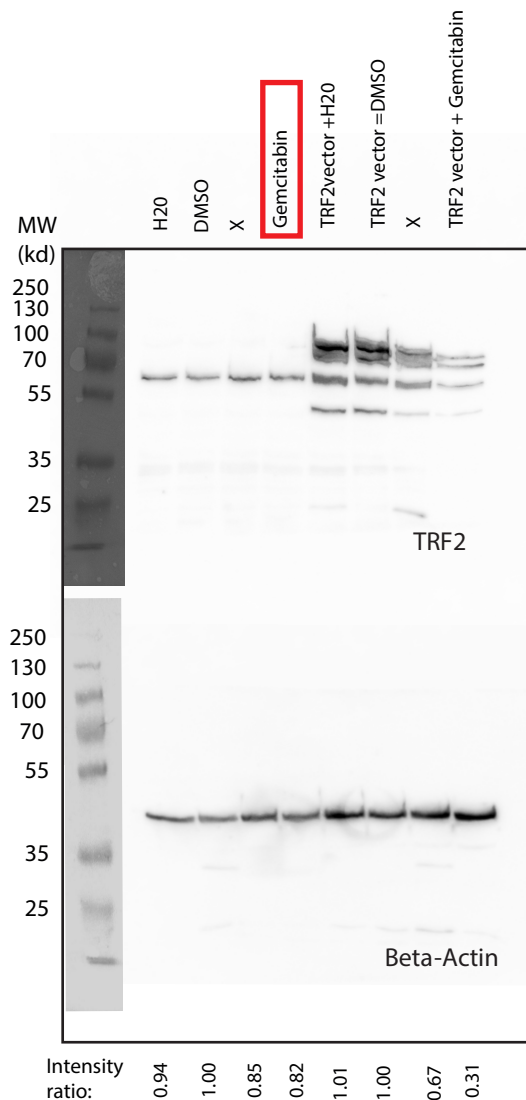

B

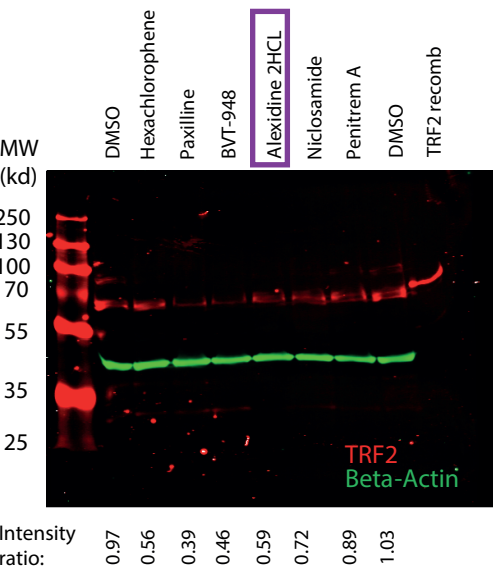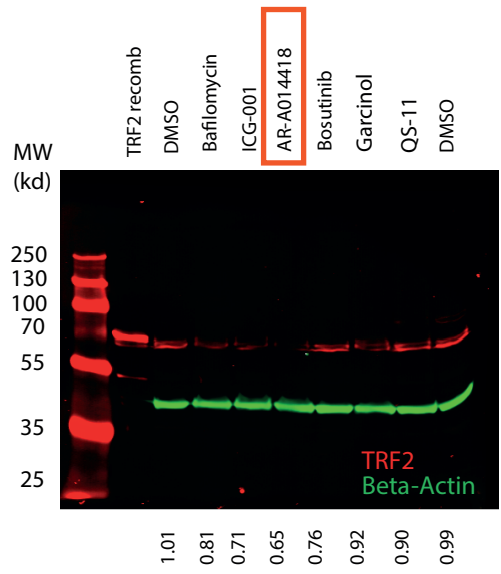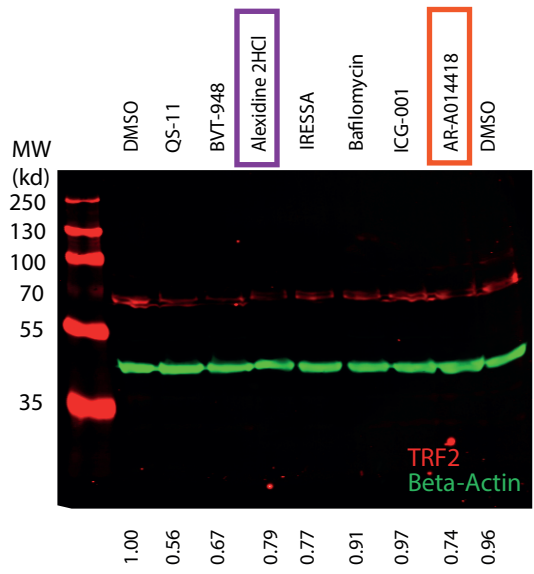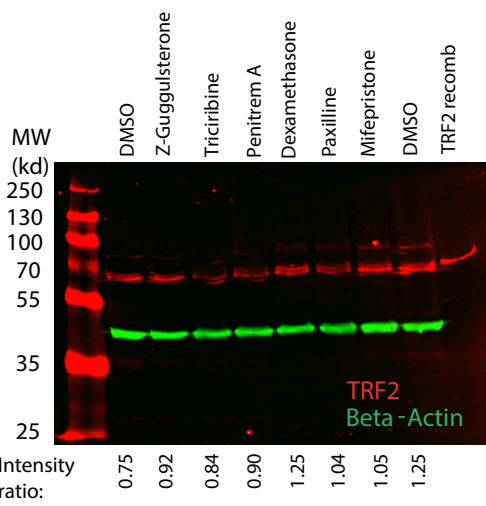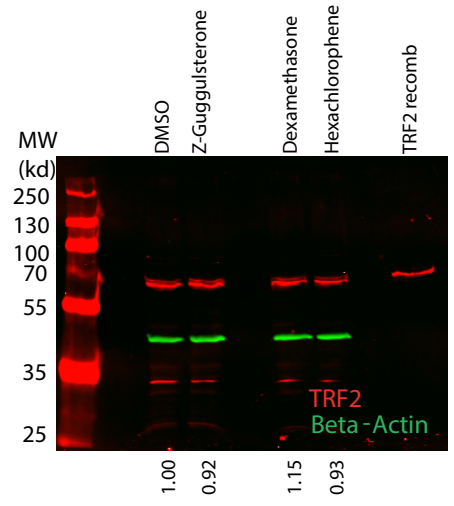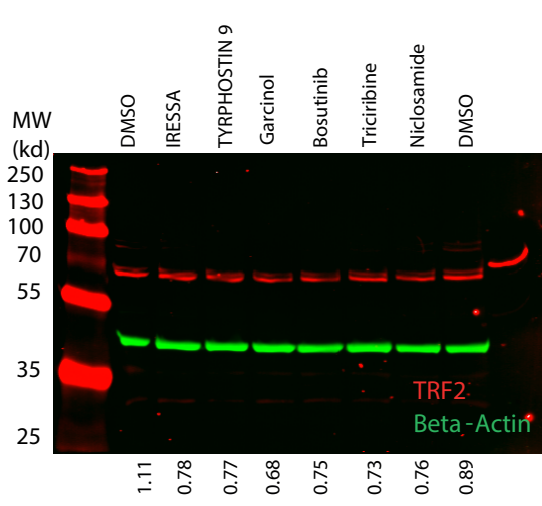

Supplement: Supplementary file 1 [file cancers-13-02998-s001.zip › 9_originalBlots.pdf]
